# Transcriptome profile of lung dendritic cells after in vitro porcine reproductive and respiratory syndrome virus (PRRSV) infection

**DOI:** 10.1371/journal.pone.0187735

**Published:** 2017-11-15

**Authors:** Maren Julia Pröll, Christiane Neuhoff, Karl Schellander, Muhammad Jasim Uddin, Mehmet Ulas Cinar, Sudeep Sahadevan, Xueqi Qu, Md. Aminul Islam, Mikhael Poirier, Marcel A. Müller, Christian Drosten, Dawit Tesfaye, Ernst Tholen, Christine Große-Brinkhaus

**Affiliations:** 1 Institute of Animal Science, Department of Animal Breeding and Husbandry, University of Bonn, Bonn, Germany; 2 School of Veterinary Science, The University of Queensland, Gatton Campus, Gatton, Australia; 3 Department of Animal Science, Faculty of Agriculture, Erciyes University, Kayseri, Turkey; 4 BRIC - Biotech Research & Innovation Centre, University of Copenhagen, Copenhagen, Denmark; 5 Laboratory for Gene and Cell Engineering, Institute of Biomedicine and Biotechnology, Shenzhen Institute of Advanced Technology, Chinese Academy of Sciences, Shenzhen, China; 6 Institute of Virology, Helmut-Ruska-Haus, Charité Medical School, Berlin, Germany; Huazhong Agriculture University, CHINA

## Abstract

The porcine reproductive and respiratory syndrome (PRRS) is an infectious disease that leads to high financial and production losses in the global swine industry. The pathogenesis of this disease is dependent on a multitude of factors, and its control remains problematic. The immune system generally defends against infectious diseases, especially dendritic cells (DCs), which play a crucial role in the activation of the immune response after viral infections. However, the understanding of the immune response and the genetic impact on the immune response to PRRS virus (PRRSV) remains incomplete. In light of this, we investigated the regulation of the host immune response to PRRSV in porcine lung DCs using RNA-sequencing (RNA-Seq). Lung DCs from two different pig breeds (Pietrain and Duroc) were collected before (0 hours) and during various periods of infection (3, 6, 9, 12, and 24 hours post infection (hpi)). RNA-Seq analysis revealed a total of 20,396 predicted porcine genes, which included breed-specific differentially expressed immune genes. Pietrain and Duroc infected lung DCs showed opposite gene expression courses during the first time points post infection. Duroc lung DCs reacted more strongly and distinctly than Pietrain lung DCs during these periods (3, 6, 9, 12 hpi). Additionally, cluster analysis revealed time-dependent co-expressed groups of genes that were involved in immune-relevant pathways. Key clusters and pathways were identified, which help to explain the biological and functional background of lung DCs post PRRSV infection and suggest *IL-1β1* as an important candidate gene. RNA-Seq was also used to characterize the viral replication of PRRSV for each breed. PRRSV was able to infect and to replicate differently in lung DCs between the two mentioned breeds. These results could be useful in investigations on immunity traits in pig breeding and enhancing the health of pigs.

## Introduction

Porcine reproductive and respiratory syndrome (PRRS) is one of the most economically important viral pig diseases worldwide [1]. The PRRS virus (PRRSV) is a single-stranded 15 kb positive-sense RNA virus [2,3]. In the late 1980s, the first clinical outbreak was observed in the United States, and in 1991, the virus was isolated and named PRRSV Lelystad strain (LV) in the Netherlands [4,5]. PRRS is characterized by reproductive failure in sows and respiratory disease patterns in pigs of all ages [4,6]. PRRS leads to high financial and production losses in the global swine industry [7–9]. The control of PRRS within a pig population remains problematic due to its etiopathology, which relies on multiple factors, such as infectious agents, the host, environmental and management considerations as well as genetic factors of host and virus [10,11]. Moreover, due to the limited availability of immunologically effective vaccines, the control of PRRSV still remains problematic [12–14].

In several studies, the mechanisms by which PRRSV modulates the innate immune response by altering cytokine patterns have been discussed [15–17]. PRRSV has developed various strategies to evade the host's immune response in innate cells, such as macrophages, monocytes and dendritic cells (DCs) [5,15,16,18–22]. The innate immune response is the first line of defense and activates adaptive host defense mechanisms [23]. DCs are the important antigen-presenting cells that are responsible for the activation of adaptive immune cells and the production of cytokines and chemokines as well as playing a role as intercellular messengers [2,6,24]. These DCs are described as “gatekeepers” of the adaptive immune system (bridge between innate and adaptive immune response) [25]. Furthermore, variations in the host's susceptibility and resistance could be explained by genetic components involved in the response to the PRRSV infection [26,27]. The understanding of the genetic elements, their functions, and the reasons for their resulting ineffective immune response to PRRSV infection still remains unclear. Therefore, the objectives of this study were to investigate the transcriptome profile of lung DCs of two genetically different pig breeds (sire lines) after PRRSV infection in vitro and to examine the temporal changes in transcriptional profiles using the RNA-Seq technology. The transcriptome profiles were obtained by applying a Gene Set Enrichment Analysis (GSEA) to characterize the functional background of lung DCs post PRRSV infection and to improve the understanding of the hosts' innate immune responses. The results will help to improve the health of pigs based on the knowledge gained about the breed-dependent differences in response to PRRSV.

## Materials and methods

### Ethics statements

The research proposal was approved by the Veterinary and Food Inspection Office, Siegburg, Germany (ref. 39600305-547/15). The piglets were exposed to the same unique environmental conditions and humanely euthanized with ketamine and T61 (Pharmazeutische Handelgesellschaft mbH, Garbsen—Berenbostel, Germany). Data recording and sample collection were conducted strictly in line with German animal welfare law.

### Animals and tissues

Three Duroc and three Pietrain female piglets (30 days old) were selected at the teaching and research station in Frankenforst, University of Bonn, Germany. The piglets had been weaned and were free from all major pig diseases (PRRSV, porcine circovirus type 2, porcine parvovirus). The animals were kept and fed according to the institutional guidelines and animal husbandry regulations of Germany (ZDS) [28]. Upon euthanasia, the lungs and trachea were carefully removed and transported on ice to the lab.

### Preparation of porcine dendritic cells

Porcine lung DCs were isolated under sterile conditions as described previously [17] with some additional protocol modifications. First, the lungs were washed with sterile calcium-magnesium free GIBCO^®^ Dulbecco's Phosphate buffer saline (DPBS) (Thermo Scientific^™^, Cat #14190094). Pulmonary alveolar macrophages (PAMs) were removed by lung lavage to minimize cell contamination [29]. Cleaned lung parts were minced into small pieces in ice-cold DPBS. Afterwards, the lung pieces were poured in 50 ml tubes with DPBS supplemented with 2.5 mg of Liberase^™^ TL Research Grade (Roche, Cat #5401119001) and 20 μl of DNase I (Qiagen GmbH, Cat #79254). Incubation was carried out for 2 hours (h) in a 37°C shaking water-bath. The enzyme activity was stopped by adding lung DCs culture medium, which consisted of Roswell Park Memorial Institute (RPMI) 1640 medium (Thermo Scientific^™^, Cat #21875091) supplemented with 10% Fetal Bovine Serum (FBS) (Thermo Scientific^™^, Cat #10270106), 1% Gentamicine (10 mg/ml) (Thermo Scientific^™^, Cat #15710049), 1% Penicillin-Streptomycin 100x concentrate (Penicillin 10.000 U/ml, Streptomycin 10.000 μg/ml) (Thermo Scientific^™^, Cat #15140122), 1% Fungizone^®^ Antimycotic (2.5 μg/ml) (Thermo Scientific^™^, Cat #15290026), and 1% sodium pyruvate 100 mM (Thermo Scientific^™^, Cat #11360070). The composite was filtered through a 70 μm BD cell strainer (BD Biosciences, Cat #352350). Before the cell viability and the cell count were determined, Red Blood Cell (RBC) contamination was removed using RBC lysis buffer. Lung DCs characterization was done with flow cytometry analyses (S1 File).

### PRRSV propagation

European prototype PRRSV strain LV and mycoplasma-free cell line MARC-145 cells were donated by Prof. Dr. Nauwynck from the Department of Virology, Parasitology and Immunology, Ghent University, Belgium [3,30]. MARC-145 cells were used for the PRRSV propagation at about 1–2 days after seeding into a culture flask using Dulbecco's Modified Eagle Medium (Thermo Scientific^™^, Cat #41966052), which contained 10% FBS, 1% Penicillin-Streptomycin 100x concentrate, and 1% Gentamicine in a humidified 5% CO_2_ atmosphere at 37°C. The cytopathic effect was performed after 5–6 days post infection (dpi), and the culture supernatants were collected for use in the plaque assay.

### PRRSV-infected lung DCs

Pietrain and Duroc lung DCs were infected individually for each animal. Lung DCs were seeded in 24-well plates and incubated until the monolayer was confluent. At that moment, the lung DCs were infected with PRRSV LV at a multiplicity of infection of 0.01. PRRSV inoculum in 200 μl of Optipro serum-free medium (OptiPRO^™^ SMF) (Life Technologies GmbH, Cat #12309019) was added to each well. Furthermore, 200 μl of OptiPRO^™^ SMF without PRRSV was placed in the non-infected wells as control (0 h) (S1 Fig). After 1 h of incubation at 37°C in 5% CO_2_, all medium from wells was aspirated, and the cells were washed with DPBS. Subsequently, 500 μl of lung DCs culture medium was added to each well. Lung DCs and the cell culture supernatant were harvested at six different experimental time-points (before (0 h) and 3, 6, 9, 12, and 24 hours post infection (hpi)).

### mRNA isolation for global transcriptome profile

Total cellular mRNA from non-infected (0 h) and infected (3, 6, 9, 12 and 24 hpi) lung DCs within the pooled Pietrain (pool n = 3) and pooled Duroc (pool n = 3) groups was extracted with the AllPrep^®^ DNA/RNA/Protein Mini Kit (Qiagen GmbH, Cat #80004) http://www.qiagen.com/products/rnastabilizationpurification/allprepdnarnaproteinminikit.aspx according to the manufacturer's recommendations with slight modifications of the chemical volumes. The mRNA quantity and quality were measured with a Nanodrop 8000 spectrophotometer (Thermo Scientific) and an Agilent 2100 Bioanalyzer (Agilent Technologies), respectively. In total, 12 lung DCs samples were sent for global transcriptome profiling done by RNA-Seq to GATC Biotech AG (Konstanz, Germany). A TruSeq RNA Sample Preparation Kit (Illumina) was used to prepare 12 RNA samples at GATC Biotech AG. The RNA-Seq library consisted of two pools, each of which included six lung DCs RNA samples. Before starting the Illumina TruSeq RNA Sample Preparation Kit, 10 μl of Rnase and DNase-free water were added to the lung DCs RNA to a total volume of 15 μl. The quality of the libraries was assessed using an Agilent 2100 Bioanalyzer (GATC Biotech AG) based on the RNA integrity number (RIN). RIN between 9.30 and 10 were reported for the lung DCs RNA libraries. Briefly, the RNA-Seq processing was done with the Low-Throughput (LT) Protocol selected from the TruSeq^™^ RNA Sample Preparation Guide. An Illumina Truseq PE Cluster Kit V3 and Illumina TruSeq SBS V3 Kit were used for sequencing. The deep sequencing was performed on an Illumina HiSeq2000 machine with 100 bases in single-read mode. Initial read processing of reads from the Illumina HiSeq2000 were processed using Illumina CASAVA Pipeline Version 1.8.0 software.

### Data analyses

#### Data processing, quality check, and sequence alignment

The quality of the sequenced reads was tested with the FastQC tool [31,32]. The analysis included basic statistics, such as the sequence quality per base, sequence quality scores, base sequence content, base GC content, sequence GC content, base N content, sequence length distribution, sequence duplication levels, overrepresented sequences, and Kmer Content. In the next step, all overrepresented sequences and adapter sequences were trimmed using cutadapt software [32,33] with an error rate fixed at 5%, and overlapping rate fixed at 80%. Based on the first quality control, the first 15 bp from the raw sequence data were removed with the seqtk tool [34]. The cutadapt software removed sequence pieces that were lower than 50 bp and trimmed the sequence part with pHRED score lower than 20 (-q20). FastQC was finally used to control the filter steps and the conclusive data quality.

The alignment of the reference genome sequence set *Sus scrofa* 10.2 [35] was performed with TopHat [36], which is an efficient read-mapping algorithm designed to align reads and makes substantial use of the tool Bowtie 2 [37,38]. The SAMStat program [39] was used to display all statistics for mapped and unmapped reads. The toolset bedtools [40] and gene information from Entrez Gene ID [41] were used to display an expression table of all 12 samples after RNA-Seq.

Furthermore, the virus sequence alignment was performed with the complete LV strain genome (GenBank: M96262). This sequence mapping permits the identification of virus absence or presence in all 12 lung DCs samples and to determine virus growth during PRRSV infection. In the following step, the mapped and unmapped reads of the alignment of the reference genome were used for the viral strain alignment using the mapping tool Bowtie 2.

#### Normalization and differentially expressed gene analysis

The read count dataset was normalized using the DESeq Bioconductor package [42] in R Project, this calculation based on a DESeq estimated size factor and the size factor function [43]. Genes were excluded from further analysis if they showed a read value of 0 for both breeds at non-infected (0 h) and infected time points (3, 6, 9, 12, and 24 hpi). After that, differentially expressed genes were further determined with the DESeq Bioconductor package. Differentially expressed genes were detected by an expression pairwise contrast between PRRSV infected (3, 6, 9, 12, and 24 hpi) and non-infected cells (0 h). The statistical criteria were defined by a log2 fold change ≥ 1 or ≤ -1, p ≤ 0.05, and False Discovery Rate (FDR) of 10%.

#### Analyses of clusters, pathway enrichment, and gene ontology

All genes were selected for the cluster and network analyses using the D-NetWeaver Software [44]. Using this software, clusters of individual genes were grouped if their expression pattern was similar during the whole experiment (before 0 h, and 3, 6, 9, 12, as well as 24 hpi). The gene expression data modeling was done with mclust algorithms provided in R. This process started with the modulation of ‘mean curves’ for each cluster of genes with a smoothing spline. The Bayesian Information Criterion (BIC) represented the final number of clusters.

Functional annotation analysis of all clusters was performed using a hyper geometric gene set enrichment test in R. The Bioconductor packages biomaRt [45], org.Ss.eg.db: Genome wide annotation for Pig [46], GSEABase [47], and GOstats [48] were used for the pathway enrichment and Gene Ontology (GO) analyses. In this process, overrepresented gene sets were defined by the Kyoto Encyclopedia of Genes and Genomes database (KEGG) [49] or by the GO database and were tested using Fisher's exact test. The GO terms biological processes (BP), cellular components (CC) and molecular functions (MF) which reached statistical significance (p ≤ 0.05) were selected for the following investigations. Additionally, multiple testing corrections were preformed using FDR estimation of Benjamini-Hochberg and the Bonferroni correction (Bon. Adjusted p-values). Finally, the differently expressed genes detected for both breeds were grouped in the identified clusters.

## Results and discussion

### Transcriptome profile analysis post PRRSV infection

In the present study, the RNA-Seq technique was used for the first time to characterize transcriptional changes after PRRSV infection in Pietrain and Duroc lung DCs. The RNA-Seq analysis obtained a total number of reads between 20.9 and 30.2 million for each library and between 12.9 to 29.5 million reads after all filtration steps. In total, 74.8% to 81.3% of the read counts mapped with the reference *Sus scrofa* genome, while 18.7% to 25.2% were identified as unmapped read counts.

Previous studies used the RNA-Seq technology to investigate PAMs and lung tissue post PRRSV infection [15,50], but no analysis had been performed with lung DCs from two different pig breeds!

PRRSV has multiple strategies to evade and modulate the host immune response. Immunomodulation post PRRSV infection resulted in inhibited cell-mediated immune reactions [16,19,20,29]. Lunney et al. [51] postulated that dysregulated expression of immune genes post PRRSV infection leads to a weakened adaptive immune response. DCs play the role of “gatekeepers” of the adaptive immune system [25]. The antigen processing and presentation of DCs and their crucial role in the secretion of inflammatory cytokines ultimately induce the immune response [52]. Rodriguez-Gomez et al. [53] revealed that an interaction between PRRSV infection and antigen-presenting cells (APCs) lead to an inaccurate or a non-effective immune response.

In the present study, a total of 20,396 porcine predicted genes were determined, and were involved in different signaling cascades affected by PRRSV infection. The temporal (3, 6, 9, 12, and 24 hpi) transcriptome profiles of PRRSV infected lung DCs of the two different breeds could be defined. These predicted genes and their differential expression post PRRSV infection were identified and interpreted.

### PRRSV infection and viral replication in lung DCs

**Fig 1:**
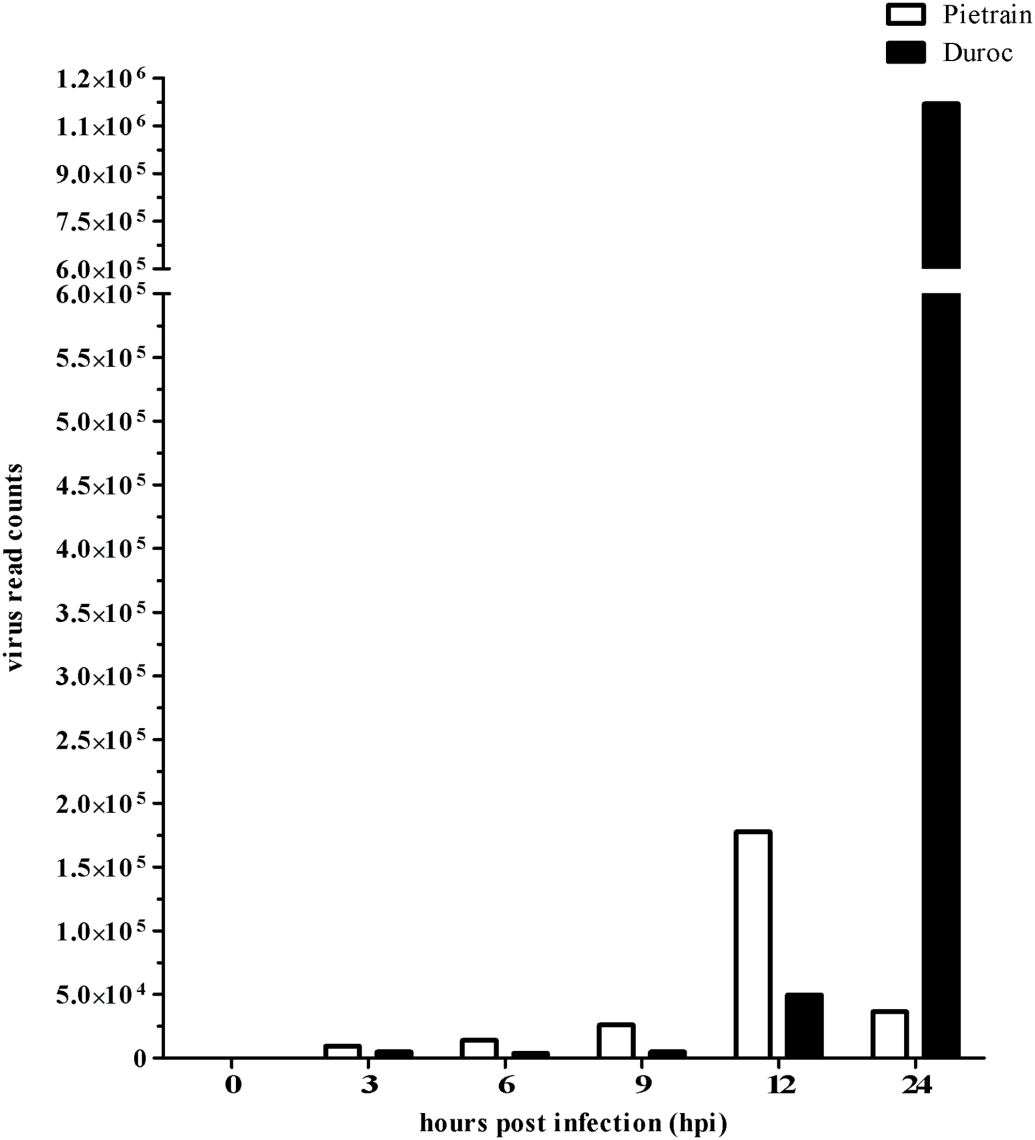
Lelystad virus (LV) growth in lung DCs. Virus read counts in Pietrain (white bar) and Duroc (black bar) lung DCs before (0 h) and post (3, 6, 9, 12, 24 hpi) PRRSV infection.

The aim of the LV strain sequence alignment was to identify the presence or absence of PRRSV in non-infected (0 h) and infected (3, 6, 9, 12, and 24 hpi) lung DCs (Fig 1). As expected, none were found in non-infected cells (0 h). Moreover, it was remarkable that the virus growth differed over all infection time points for lung DCs of Pietrain and Duroc piglets. An exponential increase (greater than 17.8-fold) was found for Pietrain lung DCs beginning at 3 hpi and ending at 12 hpi. Interestingly, the virus read counts decreased considerably for Pietrain lung DCs between 12 and 24 hpi. In contrast, the read counts for Duroc at 3, 6, and 9 hpi remained constant. But at 12 hpi the read counts for Duroc lung DCs increased substantially (greater than 21.5-fold) until 24 hpi (Fig 1).

The presence of PRRSV in infected lung DCs is a contradiction to the previous findings of Loving et al. [17]. Loving et al. [17] described that PRRSV did not replicate in lung DCs and concluded that PRRSV utilizes lung DCs without a viral replication. In the present study, the complete genome alignment results of the LV strain indicated that PRRSV LV was able to infect lung DCs and to replicate there. One reason for these different observations might be differences in the virus strains used.

In the present study breed differences were detected in relation to the measured viral read amounts with respect to important changes (Fig 1). Time course analysis of the viral replication also revealed that time point 12 hpi was very important for both breeds and only time point 24 hpi was specific for Duroc. It can be supposed that crucial molecular changes in the cells happened at these two different time points in such a way that the virus replication for Pietrain decreased until 24 hpi and increased extremely for Duroc until 24 hpi (Fig 1). The reasons for these different reactions may be that PRRSV modulated the host immune responses and the virus grew intermittently due to an early apoptosis reaction of the lung DCs of Pietrain.

It is well known that some viruses can repress apoptosis and induce host cell cycle arrest to gain more time to exploit the cells for replication [15,54,55]. Further research is needed to clarify these cell cycle related processes in PRRSV infected lung DCs. We hypothesize that these breed differences might also be related to an early immunomodulation process of PRRSV. Concerning the virus replication, Duroc lung DCs reacted more strongly and distinctly than Pietrain lung DCs during these periods of infection (3, 6, 9, and 12 hpi).

### Virus-host interaction

**Fig 2:**
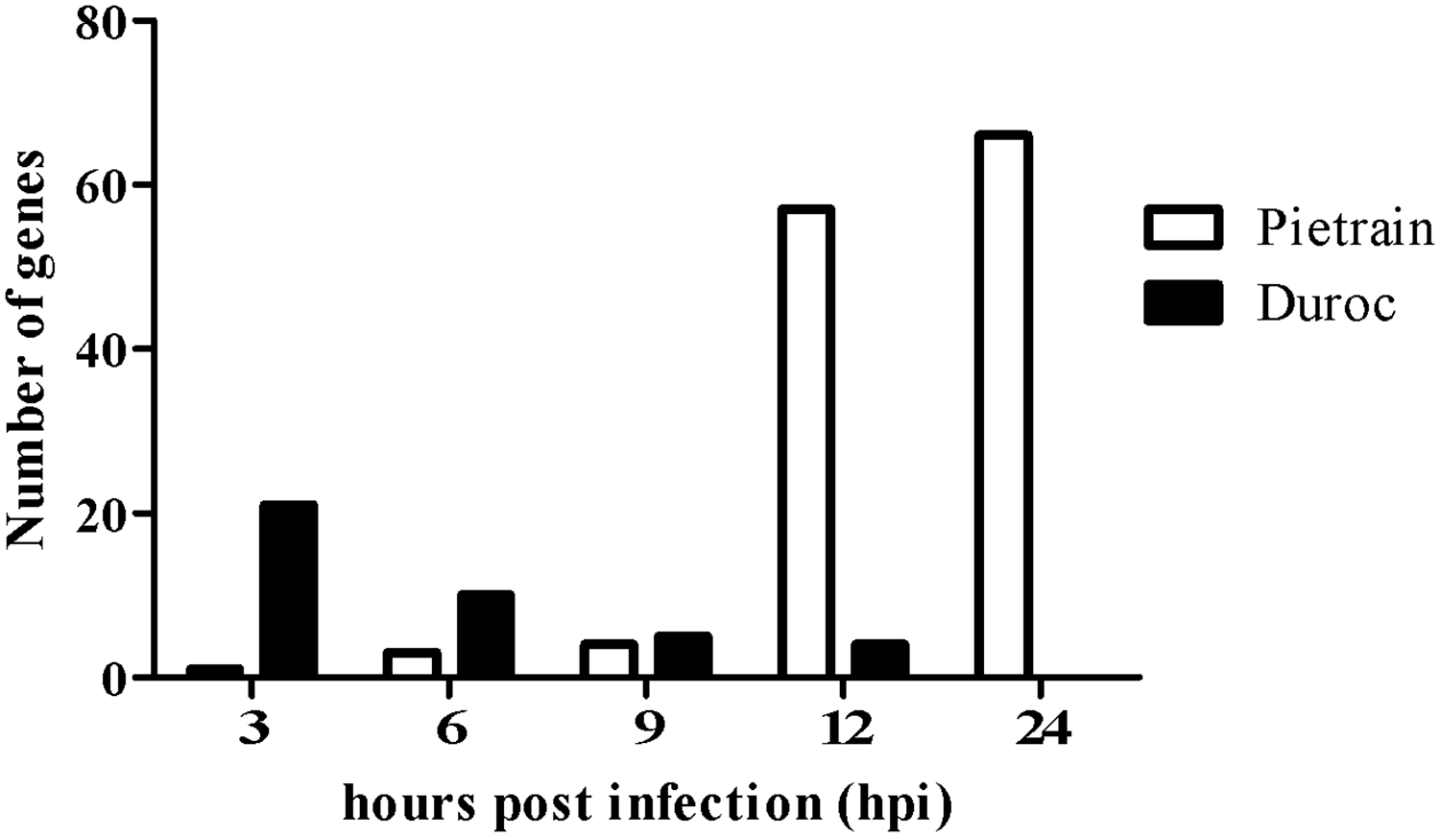
Number of down-regulated genes during the course of PRRSV infection. Pietrain (white bar) and Duroc (black bar) lung DCs at 3, 6, 9, 12, 24 hpi (p ≤ 0.05 and FDR ≤ 10%).

**Fig 3:**
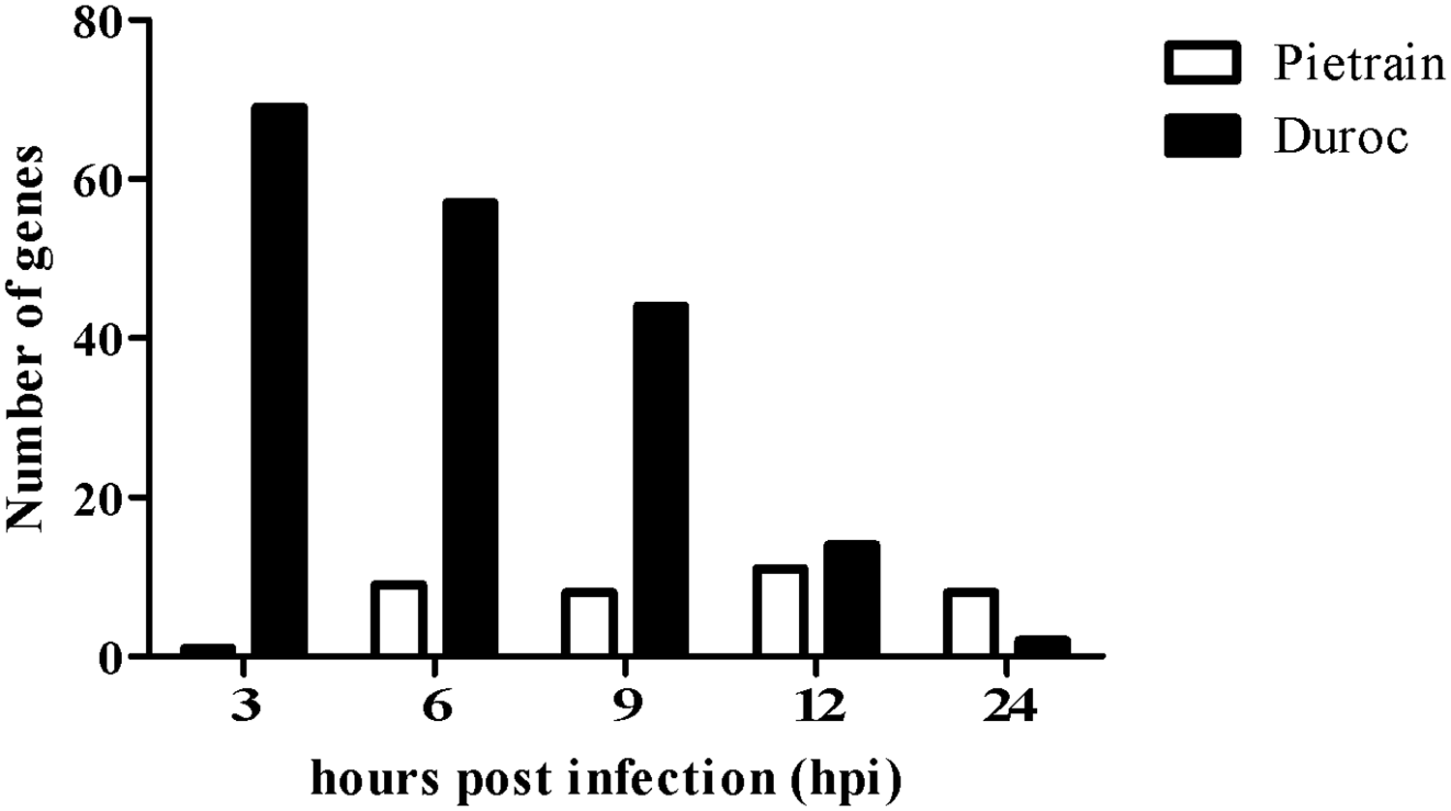
Number of up-regulated genes during the course of PRRSV infection. Pietrain (white bar) and Duroc (black bar) lung DCs at 3, 6, 9, 12, 24 hpi (p ≤ 0.05 and FDR ≤ 10%).

The pairwise comparisons between non-infected (0 h) and PRRSV infected lung DCs (3, 6, 9, 12 and 24 hpi) in two breeds were carried out to determine the effects of PRRSV infection on the host transcriptome profile. The differently expressed genes (p ≤ 0.05) between breeds were presented with respect to the FDR in two categories: those with FDR ≤ 10% and those with FDR > 10%. The analyses obtained a total of 4472 differentially expressed genes (p ≤ 0.05, FDR > 10%) in PRRSV infected lung DCs. In total, 168 genes were differentially expressed (p ≤ 0.05, FDR ≤ 10%) in Pietrain PRRSV infected lung DCs. Among them, 131 showed a down-regulation (Fig 2), and 37 genes showed an up-regulated (Fig 3) gene expression profile. For infected Duroc lung DCs, a total of 227 differentially expressed genes were identified (p ≤ 0.05, FDR ≤ 10%). Among them, 40 genes exhibited a down-regulated gene expression trend (Fig 2), and 187 showed an up-regulated trend (Fig 3). Pietrain and Duroc infected lung DCs showed opposite gene expression courses. At the early stage of infection (3 hpi), Pietrain lung DCs showed a smaller number of down-regulated genes, followed by an extreme increase until 24 hpi. In contrast, the Duroc lung DCs showed a high up-regulation at 3 hpi, followed by an extreme reduction of the gene expression until 24 hpi. In detail, PRRSV induced a remarkable increase of down-regulated immune genes (*CXCL2*, *IL-6*, *IL-1β1*, *TNF*, *CCL4*, *IL-1α*, *SLA-DRA*, *CCL3L1*, *CCL23*, *CCL20*) for infected Pietrain lung DCs from 9 to 24 hpi (Fig 2), whereas a decline was observed within infected Duroc lung DCs. For infected Duroc lung DCs a considerable decrease in up-regulated immune genes (*CCL4*, *CXCL2*, *IL-1β1*, *CXCL10*, and *CCL8*) was detected from 3 to 24 hpi (Fig 3) and the number of up-regulated genes remained constant for infected Pietrain lung DCs (Fig 3).

In Figs 2 and 3 it is clear that there were strong breed-specific gene expression differences at early time points post PRRSV infection. An early host transcriptional variation occurred during the PRRSV infection between 3 and 24 hpi. These early changes differed between Duroc and Pietrain PRRSV infected lung DCs and revealed differences in response to PRRSV infection. Similarly, Genini et al. [16] and Ait-Ali et al. [56] reported that notable reactions could be observed in pig cells (Rattlerow–Seghers genetic line, Landrace and Pietrain, respectively) within 24 h post virus infection, particularly between 8 and 10 hpi. Ait-Ali et al. [56] interpreted this time to be critical when PRRSV evaded the host responses and enhanced its own chances for viral growth. Genini et al. [16] observed a consistent down-regulation of genes after a few hours of PRRSV infection (3 and 6 hpi), followed by the start of innate immune responses at 9 hpi. Other authors had also identified variations in the host's susceptibility and resistance, which might be explained by genetic components involved in the response to PRRSV infection [26,27,57]. Concerning the up- and down-regulated expression of Duroc and Pietrain immune genes, Duroc responded better in the first time points of infection (3, 6, 9, 12 hpi) than Pietrain.

In the present study, differentially expressed genes (for example *IL-1β1*, *CCL4*, *CXCL2*) showed massive changes and contrary responses to PRRSV after a few hours of infection. Several studies [15,16,18,58] reported that PRRSV has the ability to escape or modulate the host immune system by inhibiting and inducing expression of regulatory cytokines and chemokines. These cytokines and chemokines function as mediators of the immune system, and they are known to interact with other immune cells as well as to induce inflammatory and adaptive immune responses [23,59]. For instance, *Interleukin 12* (*IL-12)* and *IL-6* stimulate the JAK-STAT signaling pathway by binding with their receptor. In turn, this pathway activates the expression of IFN-stimulated genes (ISGs) [59–61]. Furthermore, *IL-6* signaling activates the Signal transducer and activator of transcription 3 (*STAT3*), and plays an anti-inflammatory role [6,23,60]. Chemokines constitute a family of at least 50 chemoattractants that are involved in the course of many inflammatory responses. They coordinate the migration of cells and play an important role in the selective recruiting of monocytes, neutrophils, and lymphocytes [6,60]. *Chemokine (C-C motif) ligand 4* (*CCL4)* is an important chemoattractant that plays a role in viral diseases [62–64]. *Chemokine (C-X-C motif) ligand 2* (*CXCL2)* is a pro-inflammatory chemokine and attracts neutrophils as well as T cells [63–65]. *Interleukin 1*, *beta 1* (*IL-1β1)* is a major mediator of the innate immune response and induces the expression of many genes in different cells [66–68]. *IL-1β* is secreted by blood monocytes, tissue macrophages, DCs, B lymphocytes, and natural killer (NK) cells [69].

In summary, the different genetic backgrounds of the two breeds might explain the increasing number of down-regulated immune genes of infected Pietrain lung DCs from 9 to 24 hpi and the decreasing number of up-regulated immune genes of Duroc lung DCs between 3 and 24 hpi. Additionally, essential processes occurred at 9 hpi with drastic changes in the host expression trends of Duroc and Pietrain lung DCs. As mentioned above, it is well known that a remarkable number of genes are involved in the early cell-mediated immune response post PRRSV. Concerning the up- and down-regulated expression courses of Pietrain and Duroc immune genes, Duroc lung DCs reacted more actively and distinctly compared to Pietrain lung DCs at the first time points of virus infection. These observed down-regulated expression trends and postulations by Rodriguez-Gomez et al. [53], Wang et al. [70], Park et al. [71] suggest that Pietrain lung DCs were not able to activate the following gene cascades or to stimulate other immune reactions. The result is a non-effective immune response to PRRSV infection.

### Functional analyses of the clustered genes

In order to characterize the genetic background of the effects of PRRSV infection in both breeds, the 20,396 genes were condensed via cluster analysis based on the similar expression patterns during the experiment (before infection (0 h) and 3, 6, 9, 12, and 24 hpi). The clustering is solely based on the mean gene expression level for the identified genes for both breeds. The cluster construction presented 37 different transcriptional reactions to PRRSV for Pietrain lung DCs (37 cluster) and 35 different transcriptional responses to PRRSV for Duroc lung DCs (35 cluster). A GSEA of these clusters was performed to investigate the possible viral influences on the gene expression and to uncover the interaction between the virus with the host immune response. Using pathway enrichment analysis, the biological response post PRRSV infection can be characterized through the identified clusters and genes involved in the pathways for each breed.

The pathway enrichment analysis was performed for all clusters of Pietrain and Duroc, revealing a total of 171 pathways (p < 0.05) for both breeds, of which additional 47 pathways (p < 0.05) were found to be specific for the Duroc breed. The pathways of particular interest were those which reached the statistical significance (p ≤ 0.05) and showed clusters with read counts over 20. The second criterion for the selection of pathways was the frequency of occurrence for each breed. Tables 1 and 2 include the cross-classified information about the 10 most important pathways and clusters that had a significant impact on these pathways. Each cluster can be identified by a breed-specific cluster ID. In addition, the sum of genes within and across the clusters and pathways are also presented. The sum of clusters for each pathway and the cluster frequency are shown in Tables 1 and 2. Some clusters are only significant for pathways that were not ranked in the top 10, so they were not included in these tables.

**Table 1:** Top 10 scored pathways using KEGG and clustered RNA-Seq dataset post PRRSV infection, represented by the cluster ID of Pietrain lung DCs.

Pathway	Pietrain cluster IDs																								
	9	10	12	14	15	16	17	18	21	22	24	25	27	28	29	30	31	32	33	34	35	36	37	Σ cluster per pathway	Σ genes per pathway
**Phagosome** **(KEGG ID 4145)**			6							5									17	12	8	11	2	**7**	**61**
**Spliceosome** **(KEGG ID 3040)**														8	7		10	6		16				**5**	**47**
**Protein processing in endoplasmic reticulum** **(KEGG ID 4141)**														10			17		13	23	11			**5**	**74**
**Focal adhesion** **(KEGG ID 4510)**															10		10				12	11	5	**5**	**48**
**Endocytosis** **(KEGG ID 4144)**														10				7		21				**3**	**38**
**Rheumatoid arthritis** **(KEGG ID 5323)**					5										6						6	5		**4**	**22**
**Homologous recombination** **(KEGG ID 3440)**				2			3	3			2													**4**	**10**
**Cell cycle** **(KEGG ID 4110)**						8		6	6			5												**4**	**25**
**Oxidative phosphorylation** **(KEGG ID 190)**													6			19			25	15				**4**	**65**
**Jak-STAT signaling pathway** **(KEGG ID 4630)**	4	7			7													7						**4**	**25**
**Σ cluster frequency**	1	1	1	1	2	1	1	2	1	1	1	1	1	3	3	1	3	3	3	5	4	3	2		

**Table 2:** Top 10 scored pathways using KEGG and clustered RNA-Seq dataset post PRRSV infection, represented by the cluster ID of Duroc lung DCs.

Pathway	Duroc cluster IDs																					
	1	3	6	7	18	19	20	22	23	25	26	27	28	29	30	31	32	33	34	35	Σ cluster per pathway	Σ genes per pathway
**Phagosome** **(KEGG ID 4145)**										7					6	10	12	9	9		**6**	**53**
**Spliceosome** **(KEGG ID 3040)**								11	7					10			14				**4**	**42**
**Protein processing in endoplasmic reticulum** **(KEGG ID 4141)**													15	15		12	14				**4**	**56**
**Focal adhesion** **(KEGG ID 4510)**											11							9	9	3	**4**	**32**
**Endocytosis** **(KEGG ID 4144)**		5						8		8					9		14				**5**	**44**
**Rheumatoid arthritis** **(KEGG ID 5323)**										5					6		7		5		**4**	**23**
**Homologous recombination** **(KEGG ID 3440)**	4		3		4	3															**4**	**14**
**Cell cycle** **(KEGG ID 4110)**	9		6		6	9															**4**	**30**
**Oxidative phosphorylation** **(KEGG ID 190)**												14	8			20	15				**4**	**57**
**Jak-STAT signaling pathway** **(KEGG ID 4630)**				6			4														**2**	**10**
**Σ cluster frequency**	2	1	2	1	2	2	1	2	1	3	1	1	2	2	3	3	6	2	3	1		

The results of the pathway analyses show multiple occurrences of specific clusters, such as clusters 28, 32, 33, 34, and 35 for the Pietrain breed (Table 1). The results were similar for some of the Duroc clusters, while some clusters (clusters 30, 31, 32 and 34) occurred more frequently (Table 2). Especially, Pietrain cluster 34 (five occurrences) and Duroc cluster 32 (six occurrences) had the highest pathway frequencies (Tables 1 and 2, S3–S6 Tables).

In addition, the top 10 scored pathways for both breeds included phagosome (KEGG ID 4145), spliceosome (KEGG ID 3040), endocytosis (KEGG ID 4144), and JAK-STAT signaling (KEGG ID 4630) pathways. These key pathways are involved in specific functional tasks that are important for PRRSV infected DCs and for virus-host interaction (Tables 1 and 2). For example, Nauwynck et al. [72] demonstrated that the receptor mediated endocytosis might be a common entry route for arteriviruses, and De Baere et al. [73] mentioned the importance of the phagocytosis pathway for the virus-host interplay. Findings of Nauwynck et al. [72] and De Baere et al. [73] were confirmed by our results. Chen et al. [20] demonstrated that the viral NSP1β inhibits the phosphorylation and activation of *STAT1* in the JAK–STAT signaling pathway, highlighting the importance of the JAK–STAT signaling pathway during virus-host interplay. According to Lunney et al. [51], understanding the signaling pathways and molecular details could provide a valuable therapeutic opportunity and lead to clinical trials.

**Fig 4:**
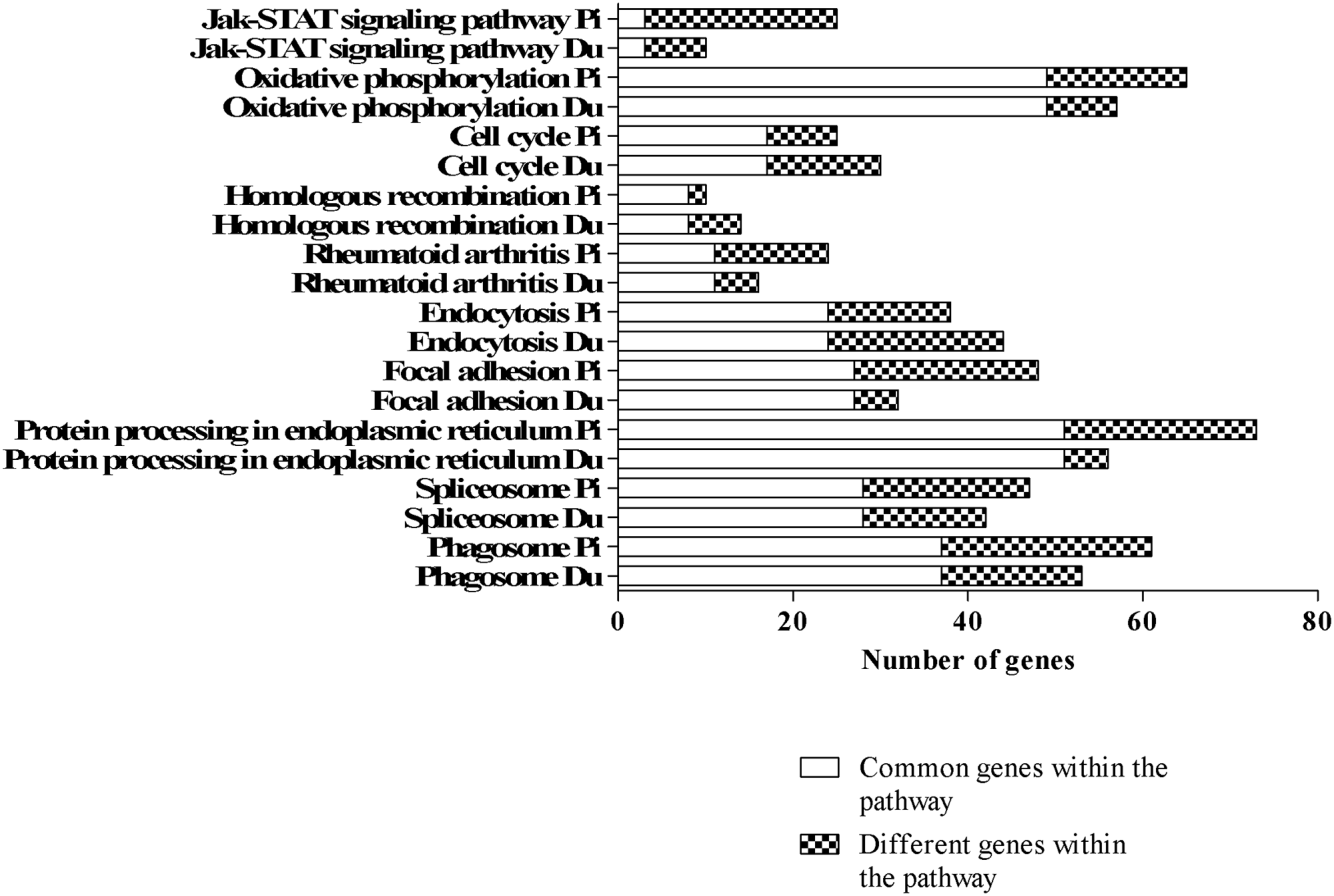
Number of genes per pathway. The numbers of genes are on the x-axis for the top 10 scored pathways of Pietrain (Pi) and Duroc (Du) lung DCs. Common genes within the pathway for each breed are depicted as a white bar and different genes are shown as a checked bar.

We also analyzed potential pathways (Tables 1 and 2) and their relevant genes (Fig 4). In summary, the amount of genes varied between breeds, which can be explained by the different numbers of clusters and cluster-specific genes (Fig 4). The phagosome pathway had the highest occurrence of clusters with seven for Pietrain and six for Duroc (Tables 1 and 2), which contain 61 and 53 genes, respectively (Fig 4). The pathway associated with protein processing in endoplasmic reticulum showed the highest number of genes for Pietrain lung DCs and the oxidative phosphorylation pathway for Duroc (Fig 4). The lowest number of genes occurred in the homologous recombination pathway for Pietrain and the JAK-STAT signaling pathway for Duroc (Fig 4).

In order to describe the genes related to the top 10 pathways and breeds, we comparatively analyzed the genes' frequency between Pietrain and Duroc (Tables 1 and 2, Fig 4). The genes selected had to be within more than one of the top 10 pathways of both breeds. The results showed that pathways of both breeds featured the genes *ATP6V1G1*, *ATP6V1C1*, and *ATP6V1F*, *SEC61B* as well as *SEC61A1*. Furthermore, *SLA-7* and *RAB5C* were found in the same pathways—endocytosis and phagosome—for Pietrain (cluster 34) and Duroc (cluster 32). The genes *ATP6V1B2*, *ATP6V1F* and *ATP6V1E1* were also detected in the—phagosome and oxidative phosphorylation—pathways for both breeds Pietrain (clusters 33 and 34) and Duroc (cluster 32). The *THBS2* thrombospondin 2 gene was also found for Duroc (cluster 33) and Pietrain (cluster 36) in the—focal adhesion and phagosome—pathways. There was no uniform regulation of these genes due to the genes of one pathway originating in different clusters, which have different expression trends post PRRSV infection.

As mentioned above, several genes of the v-ATPase complex were identified for both Duroc and Pietrain breeds *(ATP6V1G1*, *ATP6V1C1*, *ATP6V1B2*, *ATP6V1F*, and *ATP6V1E1)*. This group of genes belongs to the ER-resident multimeric protein complex [74]. The genes of the v-ATPase complex play roles in the entry of enveloped viruses and bacterial toxins, as well as in proton transport and in the acidification of endosomes [75]. The *ATP6V1B2* gene was previously detected in PAMs infected with HP-PRRSV WUH3 [61]. Furthermore, *SEC61β* and *SEC61A1* were found for both breeds in the present study, of which *SEC61β* was previously found to modulate the cytotoxicity of many chemotherapeutic agents and to be sensitive to the cytotoxic effects of platinum-containing drugs [74]. Our results show that the *SLA-7* gene also was detected for both Duroc and Pietrain. Hu et al. [76] mentioned that *SLA-7* is related to the swine major histocompatibility complex, especially to the non classical MHC class Ib genes. These genes seem to be suitable candidates for investigations of species-specific immunity-related roles [76]. These informations have to be linked to the postulation of Lunney et al. [51] that findings like that could help to design effective vaccines and therapeutic strategies.

The genes mentioned thus far include important functional information and play important roles in animal health issues associated with virus entry, drug performance, and species-specific immunity, which could be candidate genes post PRRSV infection.

### Functional background post PRRSV infection in key cluster 32 and key cluster 34

The biological significance of Duroc cluster 32 and Pietrain cluster 34 was extrapolated (Tables 1 and 2) and these clusters were determined as key clusters. The differentially expressed genes detected in both breeds were grouped in the particular clusters after the GO analysis. As mentioned previously, 227 differentially expressed genes were identified for Duroc lung DCs, of which cluster 32 included 17 of these genes (p ≤ 0.05, FDR ≤ 10%). Nine genes were up-regulated and two were down-regulated, while six genes showed up- and down-regulated expression trends at different time points (3, 6, 9, 12, and 24 hpi) (S3 Table). Additionally, a total of 168 differentially expressed genes were identified in Pietrain lung DCs. Among them, Pietrain cluster 34 included 14 differentially expressed genes (p ≤ 0.05, FDR ≤ 10%). Two genes were up-regulated, and 12 were down-regulated (S4 Table). It is remarkable that *Interleukin 1*, *beta 1* (*IL-1β1*) (Gene ID: 397122, Synonyms *IL-1β*) was differently expressed in these two key clusters. *IL-1β1* was down-regulated at different time points (9, 12, 24 hpi) in Pietrain lung DCs (cluster 34) and up-regulated at three time points (3, 6, 9 hpi) in Duroc lung DCs (cluster 32). This contrary *IL-1β1* expression between breeds could explain why Duroc lung DCs reacted more combatively during early PRRSV infection (3, 6, 9 hpi). *IL-1β1* is a major mediator of innate immune response and induces the expression of many genes in different cells [66–68]. *IL-1β* is secreted by blood monocytes, tissue macrophages, DCs, B lymphocytes and NK cells [69]. The gene *IL-1β1* is located on *Sus scrofa* chromosom (SSC) 3 with a QTL for PRRSV susceptibility and a QTL for IL-10, Interferon gamma (IFNG), and Toll-like receptor 2 (TLR2) [77]. Uddin et al. [78] identified two QTL regions for PRRSV on SSC3: one for IFNG_PRRSV and one for IL-10_PRRSV. Additionally, in regard to the importance of *IL-1β1* in Duroc cluster 32 and Pietrain cluster 34, the value of this gene is enhanced based on its location on SSC3, where a high density of QTL for immune response and PRRSV are located, but also for its impact in breeding strategies.

Furthermore, a GO analysis was performed on Duroc cluster 32 and Pietrain cluster 34 post PRRSV infection. The GO terms consist of biological processes (BP), cellular components (CC), and molecular functions (MF) enrichment analyses for the sets of clustered RNA-Seq genes. In total, Duroc cluster 32 was involved in 463 GO BP, 111 GO CC and 110 GO MF categories, while Pietrain cluster 34 had 533 GO BP, 113 GO CC, and 158 GO MF categories, by applying the Benjamini-Hochberg procedure (FDR). The Bonferroni correction appears a bit conservative, but we included the values in Tables 3 and 4. It is notable that the differently expressed gene *IL-1β1* was found in 49 GO BP, in 5 GO CC categories, and one GO MF category of Duroc key cluster 32. *IL-1β1* was found in 53 GO BP categories, 3 GO CC categories, and in one GO MF category of Pietrain key cluster 34. *IL-1β1* was present in about 10% of the GO BP of each key cluster for each breed (10.58% of Duroc GO BP and 9.94% of Pietrain GO BP), but it was inversely expressed between the breeds. Tables 3 and 4 present the list of BP GOs for both breeds, and the additional S5 and S6 Tables represent 49 BP GOs for Duroc and 53 GO BPs for Pietrain. In total, 25 of the identified BP GOs of Duroc cluster 32 and Pietrain cluster 34 were similar for both breeds, of which include cytokine-mediated signaling pathway (GO:0019221), positive regulation of cell cycle process (GO:0090068), regulation of cell cycle (GO:0051726), and response to drug (GO:0042493). Only Duroc cluster 32 included the BP GOs of immune response (GO:0006955) and lymphocyte activation (GO:0046649) (Table 4). These GOs are strongly connected and act in important processes that influence the host immune response and cellular processes, which were consistent with previous findings by Islam et al. [79].

**Table 3:** Biological process GOs of Pietrain cluster 34 post PRRSV infection.

GO ID	Biological process	Gene Counts	P-value	Bon. Adjusted p-values	FDR
GO:0042493	Response to drug	22	0.040	1	0.045
GO:0019221	Cytokine-mediated signaling pathway	22	0.022	1	0.038
GO:0090068	Positive regulation of cell cycle process	18	0.000	0.079	0.000
GO:0042345	Regulation of NF-kappaB import into nucleus	4	0.034	1	0.045
GO:0031663	Lipopolysaccharide-mediated signaling pathway	4	0.034	1	0.045

**Table 4:** Biological process GOs of Duroc cluster 32 post PRRSV infection.

GO ID	Biological process	Gene Counts	P-value	Bon. Adjusted p-values	FDR
GO:0006955	Immune response	38	0.040	1	0.044
GO:0042493	Response to drug	18	0.010	1	0.023
GO:0046649	Lymphocyte activation	18	0.032	1	0.037
GO:0019221	Cytokine-mediated signaling pathway	12	0.026	1	0.036
GO:0090068	Positive regulation of cell cycle process	13	0.000	0.295	0.003
GO:0032496	Response to lipopolysaccharide	10	0.036	1	0.040
GO:0034097	Response to cytokine stimulus	17	0.020	1	0.032

To the best of our knowledge, this is the first study to use GO analysis to characterize the biological functions of the immune response post PRRSV infection in lung DCs. Based on our findings, the Duroc key cluster 32 and the Pietrain key cluster 34 are immunologically very important and should be studied further to unravel the molecular functions played by genes as well as to explain breed-related differences in immune reactions.

Earlier studies demonstrated that various cytokines and interleukins play a central role in the beginning of the innate immune response post PRRSV infection [16,80]. Flori et al. [81] showed weak to moderate heritabilities for pro-inflammatory cytokines (*IL-1β*, *IL-8*, *TNF and IL-6*). *IL-1β* is a critical mediator of inflammation and host response to infections [23]. Bi et al. [82] mentioned that PRRSV infection results in *IL-1β* production and described the pathways involved in the recognition of PRRSV and the production of *IL-1β*. The mRNA expression and secretion of *IL-1β* were significantly increased. Zhang et al. [83] suggested that PRRSV protein E is the main contributory factor for this PRRSV induced inflammasome activation. Furthermore, they also mentioned that the consequentially robust *IL-1β* production might be the main reason for eliciting the strong inflammatory response. Ross et al. [84] and Seo et al. [85] demonstrated that *IL-1β* plays an important role in the porcine conceptus elongation and the establishment of pregnancy. This multifactorial influence of *IL-1β1* in the beginning of the innate immune response as well as the establishment of pregnancy makes it a big challenge to understand the role of *IL-1β1* post PRRSV infection.

In summary, *IL-1β1* can be considered as an important candidate gene in the immune response to PRRSV infection. It is particularly relevant that the *IL-1β1* showed contrary expression responses to PRRSV in Duroc and Pietrain lung DCs. The up-regulation of *IL-1β1* is one explanation for the more efficient immune response of Duroc lung DCs to PRRSV infection. The decreased expression level of *IL-1β1* in Pietrain lung DCs in the present study is in conflict with the results from Bi et al. [82], who showed significantly increased expression and secretion of *IL-1β*. The results of the expression profile of *IL-1β1* in Pietrain lung DCs are also contrary to Zhang et al. [83], who postulated a consequentially robust *IL-1β* production post PRRSV infection. We also identified potential genes for several pathways and GO terms involved in the immune response to PRRSV infection and *IL-1β1* was the most prominent differently expressed gene.

## Conclusion

This is the first study to use GO analysis to determine the biological functions of the immune response post PRRSV infection and to identify possible reasons for the immune reaction in lung DCs of different breeds. The transcriptome profile analysis did reveal breed-specific differences in response to PRRSV infection. Duroc lung DCs responded better in the first time points of infection (3, 6, 9, 12 hpi) than Pietrain lung DCs. Another main observation is the capacity of PRRSV to infect Pietrain and Duroc lung DCs and its extremely breed-dependent replication pattern. Additionally, it was possible to identify key clusters and pathways as well as specific genes (e.g. *SEC61β*, *SLA-7*), that play important roles in animal health. These genes can be considered for further investigation to test their role in the development of effective and efficient vaccines for PRRSV. *IL-1β1* showed opposite responses to PRRSV in Duroc and Pietrain lung DCs, and is involved in Duroc key cluster 32 and Pietrain key cluster 34. The up-regulation of *IL-1β1* could explain the more efficient immune response of Duroc lung DCs to PRRSV infection. *IL-1β1* could be used as a potential candidate gene for selecting pigs that respond efficiently to PRRSV.

In conclusion, the present study provides important findings of the role of genetics in the response of animals to PRRSV, especially with respect to immune responses to PRRSV infection. These results should be taken into account for further investigations on immune traits in pig breeding and for improving the health of pigs.

## Supporting information

S1 File Flow cytometer analyses. (DOCX)

S1 Fig Experimental design for PRRSV infection. Duroc (n = 3, animal A1, A2, A3) and Pietrain (n = 3, animal A1, A2, A3) lung DCs infected with the Lelystad virus (LV). Sample collection: non-infected cells (control = green circle) at 0 h and infected cells (PRRSV = blue circle) at 3, 6, 9, 12, 24 hpi.
(TIF)

S2 Fig Expression profiles of *IL-1β1*. Gene expression profiles of *IL-1β1* in non-infected (0 h) and infected (3, 6, 9, 12, 24 hpi) lung DCs of Pietrain and Duroc, detected by RNA-Seq (Pietrain = circle and Duroc = square) and Real Time PCR (Pietrain = upwards triangle and Duroc = downwards triangle). *IL-1β1* was normalized with Glyceraldehyde-3-phosphate dehydrogenase (*GAPDH*) and Hypoxanthine phosphoribosyltransferase 1 (*HPRT1*).
(TIF)

S1 Table Gene IDs for Duroc cluster 32 in lung DCs. (XLSX)

S2 Table Gene IDs for Pietrain cluster 34 in lung DCs. (XLSX)

S3 Table Differently expressed genes for Duroc cluster 32 in lung DCs. (DOCX)

S4 Table Differently expressed genes for Pietrain cluster 34 in lung DCs. (DOCX)

S5 Table BP GOs for Pietrain cluster 34 in lung DCs post PRRSV infection. (DOCX)

S6 Table BP GOs for Duroc cluster 32 in lung DCs post PRRSV infection. (DOCX)
