# Supplementary material for: Transcriptome profile of lung dendritic cells after in vitro porcine reproductive and respiratory syndrome virus (PRRSV) infection
Source: PLoS One. 2017 Nov 15;12(11):e0187735. doi: 10.1371/journal.pone.0187735 (PMC5687707; doi:10.1371/journal.pone.0187735)
Supplement: S1 File — (DOCX) [file pone.0187735.s001.docx]

**Supporting Information 1 File**

**Flow cytometer analyses:**

Cell characterization by flow cytometry analyses on isolated lung DCs was performed as follows: The surface antigens antibodies were used singly and labelled with fluorescein isothiocyanate (FITC), allophycocyanin (APC), phycoerythrin and a cyanine dye 7 (PE-Cy7) as displayed in Table 2. The cells were harvested and washed with sterile DPBS. Afterwards 1 x 10^6^ cells were incubated under light protected condition with 10 μl of conjugated antibody to CD11c, CD86, CD80 and 20 μl of CD40 for 30 min at 4°C. The cells were stained, according to the antibodies manufacturers' recommendations. After two washing cycles, using the flow cytometry staining buffer (DPBS supplement with 2% FBS, 10 mM NaN3 and 10 mM HEPES) the cells were pelleted and finally resuspended within flow cytometry staining buffer. The cell characterization was performed, using the FACSCanto flow cytometer (BD Biosciences) and analysed by FlowLogic® software (BD Biosciences; Germany) in order to enumerate the cell population, based on cell surface marker.

Table 2: Antibodies, used for flow cytometry analyses

| Antibody | Isotype | Clone | Fluorescence | Company |
| --- | --- | --- | --- | --- |
| CD86 | IgG1 | 37301 | APC | R&D Systems, cat. FAB141A |
| CD80 | IgG1 | 37711 | PE | R&D Systems, cat. FAB140P |
| CD40 | IgG1 | G28.5 | FITC | NOVUS Biologicals®, cat. NB100-77786 |
| CD11c | IgG | N418 | PE-Cy7 | eBioscience, cat. 25-0114 |

The flow cytometry results showed CD11c 83%, of CD86 29%, of CD80 14% and of CD40 64% expression on the surface of lung DCs. The lung DCs exhibited the phenotypic characteristics of immature DCs, including high CD11c and low CD80/86 expression and the results, concerning the low expression of CD80 and CD86, showed an accordance with the findings of Loving et al. (2007).

**References**

Loving CL, Brockmeier SL, Sacco RE. Differential type I interferon activation and susceptibility of dendritic cell populations to porcine arterivirus. Immunology. 2007; 120: 217–229. doi: 10.1111/j.1365-2567.2006.02493.x.
