# Supplementary figures and images for: Transcriptome profile of lung dendritic cells after in vitro porcine reproductive and respiratory syndrome virus (PRRSV) infection
Source: PLoS One. 2017 Nov 15;12(11):e0187735. doi: 10.1371/journal.pone.0187735 (PMC5687707; doi:10.1371/journal.pone.0187735)

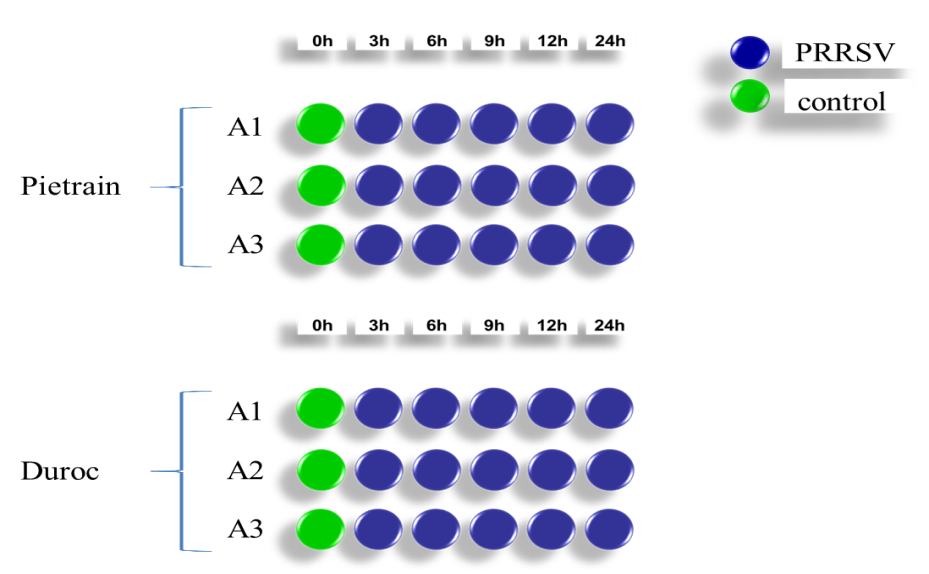

Supplement: S1 Fig — Duroc (n = 3, animal A1, A2, A3) and Pietrain (n = 3, animal A1, A2, A3) lung DCs infected with the Lelystad virus (LV). Sample collection: non-infected cells (control = green circle) at 0 h and infected cells (PRRSV = blue circle) at 3, 6, 9, 12, 24 hpi. (TIF) [file pone.0187735.s002.tif]

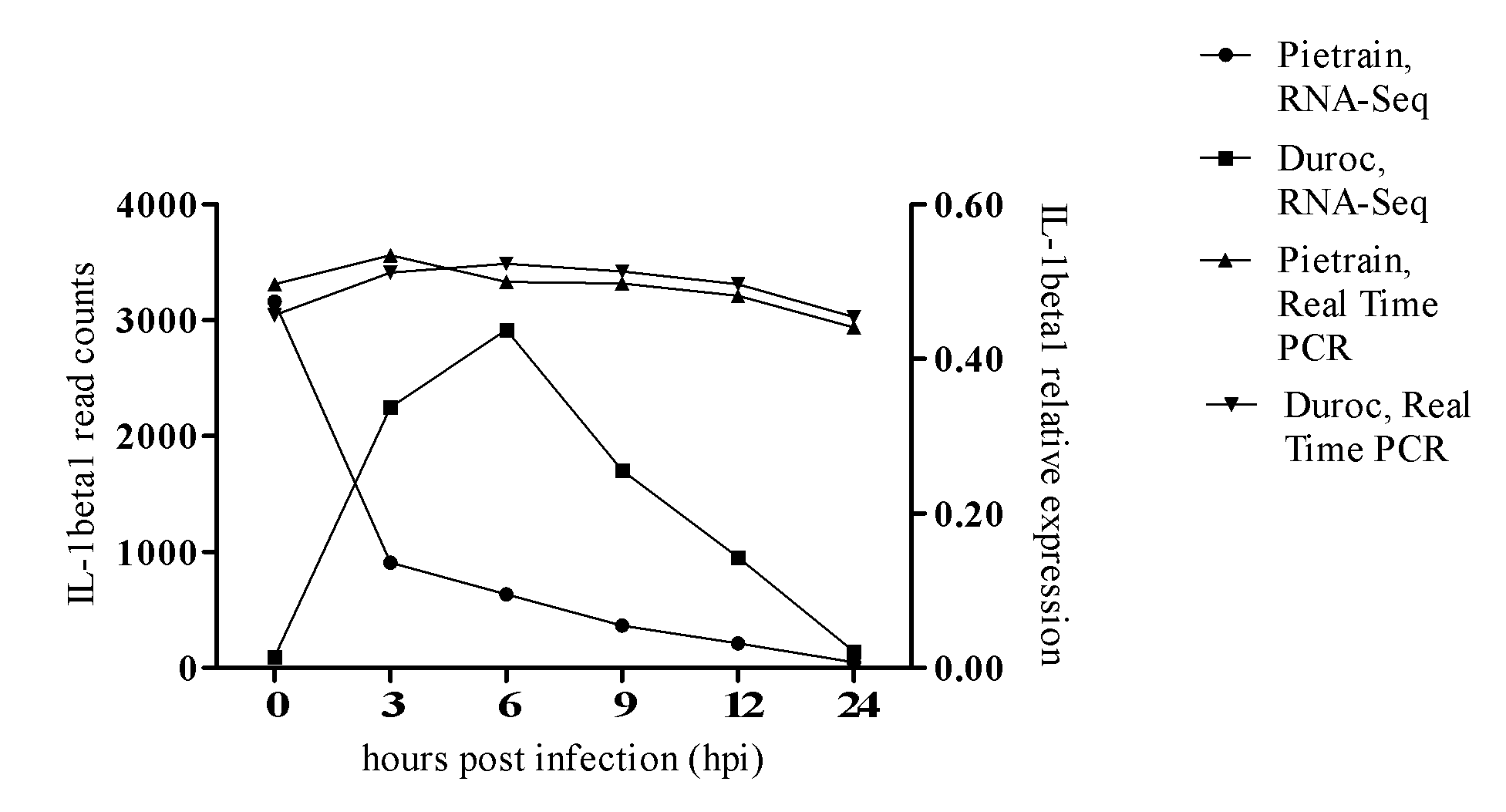

Supplement: S2 Fig — Gene expression profiles of IL-1β1 in non-infected (0 h) and infected (3, 6, 9, 12, 24 hpi) lung DCs of Pietrain and Duroc, detected by RNA-Seq (Pietrain = circle and Duroc = square) and Real Time PCR (Pietrain = upwards triangle and Duroc = downwards triangle). IL-1β1 was normalized with Glyceraldehyde-3-phosphate dehydrogenase (GAPDH) and Hypoxanthine phosphoribosyltransferase 1 (HPRT1). (TIF) [file pone.0187735.s003.tif]
