# Supplementary material for: Transcriptome profile of lung dendritic cells after in vitro porcine reproductive and respiratory syndrome virus (PRRSV) infection
Source: PLoS One. 2017 Nov 15;12(11):e0187735. doi: 10.1371/journal.pone.0187735 (PMC5687707; doi:10.1371/journal.pone.0187735)
Supplement: S3 Table — (DOCX) [file pone.0187735.s006.docx]

**S3 Table. Differently expressed genes for Duroc cluster 32 in lung DCs.**

| Entrezgene | Gene ID | SSC | log2Fold  Change | P-value | FDR | Hours post infection |
| --- | --- | --- | --- | --- | --- | --- |
| 100525209 | IER2 | 2 | -1.77216197 | P < 0.001 | 0.00045728 | 3 |
| 100415933 | SPACA4 | 6 | -4.14843088 | P < 0.001 | 1.51E-05 | 3 |
| 100415933 | SPACA4 | 6 | 3.41073084 | P < 0.001 | 0.00640954 | 9 |
| 100626471 | SULT2B1 | 6 | -3.13038225 | P < 0.001 | 0.00051201 | 3 |
| 100626471 | SULT2B1 | 6 | 3.28648065 | P < 0.001 | 0.00869704 | 9 |
| 396752 | IRG6 | 3 | 2.16722189 | P < 0.001 | 4.48E-05 | 3 |
| 100154248 | IFIT3 | 14 | 1.30777018 | P < 0.001 | 0.09422006 | 3 |
| 768102 | SRSF6 | 17 | 2.22244488 | P < 0.001 | 0.09293495 | 6 |
| 100738225 | LOC100738225 | 6 | -2.00363047 | P < 0.001 | 0.05379361 | 3 |
| 100738225 | LOC100738225 | 6 | 2.99630312 | P < 0.001 | 0.02347507 | 9 |
| 100626173 | FAM83E | 6 | 3.37468889 | P < 0.001 | 0.00640954 | 9 |
| 100626173 | FAM83E | 6 | -3.77186753 | P < 0.001 | 5.08E-05 | 3 |
| 100738145 | LOC100738145 | 6 | -3.05546326 | P < 0.001 | 0.00051615 | 3 |
| 100738145 | LOC100738145 | 6 | 3.23912893 | P < 0.001 | 0.01043538 | 9 |
| 100154509 | SRSF5 | 7 | 1.41101857 | P < 0.001 | 0.04606949 | 3 |
| 100154509 | SRSF5 | 7 | 2.51882887 | P < 0.001 | 0.03189167 | 6 |
| 100154509 | SRSF5 | 7 | 2.68752289 | P < 0.001 | 0.05170805 | 9 |
| 100738179 | NTN5 | 6 | 3.39602903 | P < 0.001 | 0.00640954 | 9 |
| 100738179 | NTN5 | 6 | -4.01194079 | P < 0.001 | 2.37E-05 | 3 |
| 397122 | IL1B | 3 | 4.55752143 | P < 0.001 | 7.75E-22 | 3 |
| 397122 | IL1B | 3 | 4.93320582 | P < 0.001 | 2.05E-08 | 6 |
| 397122 | IL1B | 3 | 4.1598166 | P < 0.001 | 0.00024697 | 9 |
| 100153946 | CEBPD | 4 | -1.46532389 | P < 0.001 | 0.01331731 | 3 |
| 397115 | ARG1 | 1 | 2.81966388 | P < 0.001 | 0.01290231 | 6 |
| 100622059 | KCTD11 | 12 | 1.98571767 | P < 0.001 | 5.02E-05 | 3 |
| 397120 | HAS2 | 4 | 1.30397695 | P < 0.001 | 0.06505742 | 3 |
| 100521185 | ZFAND2A | 3 | 1.51385582 | P < 0.001 | 0.01126138 | 3 |
