# Supplementary material for: Transcriptome profile of lung dendritic cells after in vitro porcine reproductive and respiratory syndrome virus (PRRSV) infection
Source: PLoS One. 2017 Nov 15;12(11):e0187735. doi: 10.1371/journal.pone.0187735 (PMC5687707; doi:10.1371/journal.pone.0187735)
Supplement: S4 Table — (DOCX) [file pone.0187735.s007.docx]

**S4 Table. Differently expressed genes for Pietrain cluster 34 in lung DCs.**

| Entrezgene | Gene ID | SSC | log2Fold  Change | P-value | FDR | Hours post infection |
| --- | --- | --- | --- | --- | --- | --- |
| 100620584 | NRG1 | 15 | 3.020143486 | P < 0.001 | 0.047087512 | 6 |
| 100620584 | NRG1 | 15 | 3.305616178 | P < 0.001 | 0.017421666 | 9 |
| 100620584 | NRG1 | 15 | 2.092909168 | P < 0.001 | 0.062318731 | 12 |
| 397084 | TGFB2 | 10 | 2.928493159 | P < 0.001 | 0.087110931 | 9 |
| 397122 | IL1B | 3 | -3.112283399 | P < 0.001 | 0.034296977 | 9 |
| 397122 | IL1B | 3 | -3.898881723 | P < 0.001 | 3.26E-07 | 12 |
| 397122 | IL1B | 3 | -5.996147964 | P < 0.001 | 8.53E-05 | 24 |
| 397417 | MT1A | 6 | -3.4201421 | P < 0.001 | 1.40E-05 | 12 |
| 397417 | MT1A | 6 | -4.447541824 | P < 0.001 | 0.012436864 | 24 |
| 397123 | MT3 | 6 | -3.244285684 | P < 0.001 | 3.18E-05 | 12 |
| 397123 | MT3 | 6 | -4.30677052 | P < 0.001 | 0.014731469 | 24 |
| 414904 | CXCL2 | 8 | -2.73744758 | P < 0.001 | 0.001171852 | 12 |
| 414904 | CXCL2 | 8 | -4.569769215 | P < 0.001 | 0.009082708 | 24 |
| 733670 | RGS2 | 10 | -2.382156095 | P < 0.001 | 0.010468373 | 12 |
| 733670 | RGS2 | 10 | -3.82013657 | P < 0.001 | 0.037169613 | 24 |
| 100301483 | S100A12 | 4 | -2.319997525 | P < 0.001 | 0.014962056 | 12 |
| 100301483 | S100A12 | 4 | -3.871995589 | P < 0.001 | 0.034812687 | 24 |
| 100048932 | BTG2 | 9 | -2.270973537 | P < 0.001 | 0.017273631 | 12 |
| 100737739 | RND1 | 5 | -2.193249006 | P < 0.001 | 0.029281508 | 12 |
| 100737739 | RND1 | 5 | -4.032428401 | P < 0.001 | 0.026622158 | 24 |
| 553951 | CCL20 | 15 | -3.881540102 | P < 0.001 | 0.037800423 | 24 |
| 100520219 | LEPREL1 | 13 | -3.820234669 | P < 0.001 | 0.038086132 | 24 |
| 397094 | IL1A | 3 | -3.511392825 | P < 0.001 | 0.086853509 | 24 |
| 100515954 | CDA | 6 | -3.468873921 | P < 0.001 | 0.098689931 | 24 |
