# Supplementary material for: Transcriptome profile of lung dendritic cells after in vitro porcine reproductive and respiratory syndrome virus (PRRSV) infection
Source: PLoS One. 2017 Nov 15;12(11):e0187735. doi: 10.1371/journal.pone.0187735 (PMC5687707; doi:10.1371/journal.pone.0187735)
Supplement: S5 Table — (DOCX) [file pone.0187735.s008.docx]

**S5 Table. BP GOs for Pietrain cluster 34 in lung DCs post PRRSV infection.**

| **GO ID** | **Biological process** | **Gene Counts** | **P-value** | **Bon. Adj. p-values** | **FDR** |
| --- | --- | --- | --- | --- | --- |
| GO:0006793 | phosphorus metabolic process | 154 | 0.000 | 0.265 | 0.002 |
| GO:0000278 | mitotic cell cycle | 47 | 0.001 | 1 | 0.007 |
| GO:0006605 | protein targeting | 40 | 0.000 | 0.105 | 0.001 |
| GO:0007067 | mitosis | 18 | 0.041 | 1 | 0.045 |
| GO:0006913 | nucleocytoplasmic transport | 30 | 0.000 | 0.008 | 0.000 |
| GO:0006606 | protein import into nucleus | 15 | 0.032 | 1 | 0.045 |
| GO:0007565 | female pregnancy | 9 | 0.041 | 1 | 0.045 |
| GO:0006996 | organelle organization | 131 | 0.000 | 0.000 | 0.000 |
| GO:0007167 | enzyme linked receptor protein signaling pathway | 49 | 0.038 | 1 | 0.045 |
| GO:0031329 | regulation of cellular catabolic process | 33 | 0.001 | 0.672 | 0.005 |
| GO:0008285 | negative regulation of cell proliferation | 30 | 0.008 | 1 | 0.021 |
| GO:0044093 | positive regulation of molecular function | 80 | 0.000 | 0.001 | 0.000 |
| GO:0044249 | cellular biosynthetic process | 215 | 0.008 | 1 | 0.021 |
| GO:0044260 | cellular macromolecule metabolic process | 286 | 0.000 | 0.000 | 0.000 |
| GO:0045184 | establishment of protein localization | 92 | 0.000 | 0.000 | 0.000 |
| GO:0070727 | cellular macromolecule localization | 90 | 0.000 | 0.000 | 0.000 |
| GO:0050790 | regulation of catalytic activity | 92 | 0.000 | 0.209 | 0.002 |
| GO:0051044 | positive regulation of membrane protein ectodomain proteolysis | 3 | 0.016 | 1 | 0.031 |
| GO:0051173 | positive regulation of nitrogen compound metabolic process | 61 | 0.027 | 1 | 0.042 |
| GO:0051726 | regulation of cell cycle | 24 | 0.002 | 1 | 0.009 |
| GO:0071702 | organic substance transport | 122 | 0.000 | 0.000 | 0.000 |
| GO:0071840 | cellular component organization or biogenesis | 233 | 0.000 | 0.000 | 0.000 |
| GO:0090304 | nucleic acid metabolic process | 162 | 0.001 | 0.729 | 0.006 |
| GO:0009059 | macromolecule biosynthetic process | 182 | 0.022 | 1 | 0.038 |
| GO:0009057 | macromolecule catabolic process | 71 | 0.000 | 0.001 | 0.000 |
| GO:0009891 | positive regulation of biosynthetic process | 65 | 0.022 | 1 | 0.038 |
| GO:0010604 | positive regulation of macromolecule metabolic process | 103 | 0.000 | 0.259 | 0.002 |
| GO:0009725 | response to hormone stimulus | 42 | 0.038 | 1 | 0.045 |
| GO:0010627 | regulation of intracellular protein kinase cascade | 41 | 0.013 | 1 | 0.028 |
| GO:0010646 | regulation of cell communication | 111 | 0.003 | 1 | 0.011 |
| GO:0016482 | cytoplasmic transport | 44 | 0.000 | 0.081 | 0.001 |
| GO:0019220 | regulation of phosphate metabolic process | 69 | 0.013 | 1 | 0.027 |
| GO:0022414 | reproductive process | 66 | 0.007 | 1 | 0.019 |
| GO:0023051 | regulation of signaling | 111 | 0.004 | 1 | 0.013 |
| GO:0031401 | positive regulation of protein modification process | 31 | 0.049 | 1 | 0.049 |
| GO:0032880 | regulation of protein localization | 20 | 0.049 | 1 | 0.049 |
| GO:0033365 | protein localization to organelle | 38 | 0.002 | 1 | 0.008 |
| GO:0035556 | intracellular signal transduction | 92 | 0.010 | 1 | 0.023 |
| GO:0042176 | regulation of protein catabolic process | 12 | 0.020 | 1 | 0.036 |
| GO:0043412 | macromolecule modification | 158 | 0.000 | 0.012 | 0.000 |
| GO:0044710 | single-organism metabolic process | 119 | 0.013 | 1 | 0.028 |
| GO:0045859 | regulation of protein kinase activity | 35 | 0.026 | 1 | 0.041 |
| GO:0051130 | positive regulation of cellular component organization | 32 | 0.004 | 1 | 0.013 |
| GO:0051704 | multi-organism process | 58 | 0.004 | 1 | 0.014 |
| GO:0080090 | regulation of primary metabolic process | 194 | 0.003 | 1 | 0.011 |
| GO:0080134 | regulation of response to stress | 38 | 0.020 | 1 | 0.035 |
| GO:1901701 | cellular response to oxygen-containing compound | 21 | 0.022 | 1 | 0.038 |
| GO:2000026 | regulation of multicellular organismal development | 54 | 0.041 | 1 | 0.045 |
