# Supplementary material for: Transcriptome profile of lung dendritic cells after in vitro porcine reproductive and respiratory syndrome virus (PRRSV) infection
Source: PLoS One. 2017 Nov 15;12(11):e0187735. doi: 10.1371/journal.pone.0187735 (PMC5687707; doi:10.1371/journal.pone.0187735)
Supplement: S6 Table — (DOCX) [file pone.0187735.s009.docx]

**S6 Table. BP GOs for Duroc cluster 32 in lung DCs post PRRSV infection.**

| **GO ID** | **Biological process** | **Gene Counts** | **P-value** | **Bon. Adj. p-values** | **FDR** |
| --- | --- | --- | --- | --- | --- |
| GO:0000278 | mitotic cell cycle | 36 | 0.000 | 0.300 | 0.003 |
| GO:0001932 | regulation of protein phosphorylation | 30 | 0.047 | 1 | 0.048 |
| GO:0002791 | regulation of peptide secretion | 9 | 0.037 | 1 | 0.041 |
| GO:0006605 | protein targeting | 27 | 0.001 | 0.870 | 0.006 |
| GO:0006793 | phosphorus metabolic process | 90 | 0.017 | 1 | 0.029 |
| GO:0006996 | organelle organization | 79 | 0.001 | 0.562 | 0.005 |
| GO:0007167 | enzyme linked receptor protein signaling pathway | 39 | 0.005 | 1 | 0.014 |
| GO:0007566 | embryo implantation | 3 | 0.045 | 1 | 0.046 |
| GO:0036211 | protein modification process | 97 | 0.004 | 1 | 0.012 |
| GO:0031329 | regulation of cellular catabolic process | 23 | 0.013 | 1 | 0.025 |
| GO:0032270 | positive regulation of cellular protein metabolic process | 36 | 0.001 | 0.721 | 0.006 |
| GO:0032502 | developmental process | 123 | 0.023 | 1 | 0.036 |
| GO:0033619 | membrane protein proteolysis | 5 | 0.005 | 1 | 0.014 |
| GO:0044093 | positive regulation of molecular function | 50 | 0.001 | 0.466 | 0.004 |
| GO:0044249 | cellular biosynthetic process | 149 | 0.007 | 1 | 0.017 |
| GO:0044260 | cellular macromolecule metabolic process | 226 | 0.000 | 0.008 | 0.000 |
| GO:0044703 | multi-organism reproductive process | 22 | 0.017 | 1 | 0.029 |
| GO:0044706 | multi-multicellular organism process | 8 | 0.048 | 1 | 0.048 |
| GO:0045184 | establishment of protein localization | 62 | 0.000 | 0.000 | 0.000 |
| GO:0045937 | positive regulation of phosphate metabolic process | 25 | 0.034 | 1 | 0.039 |
| GO:0070727 | cellular macromolecule localization | 58 | 0.000 | 0.000 | 0.000 |
| GO:0046883 | regulation of hormone secretion | 10 | 0.038 | 1 | 0.042 |
| GO:0048519 | negative regulation of biological process | 87 | 0.001 | 0.790 | 0.006 |
| GO:0050790 | regulation of catalytic activity | 60 | 0.006 | 1 | 0.016 |
| GO:0050796 | regulation of insulin secretion | 9 | 0.020 | 1 | 0.032 |
| GO:0051044 | positive regulation of membrane protein ectodomain proteolysis | 3 | 0.005 | 1 | 0.014 |
| GO:0051170 | nuclear import | 11 | 0.048 | 1 | 0.048 |
| GO:0051173 | positive regulation of nitrogen compound metabolic process | 3 | 0.017 | 1 | 0.030 |
| GO:0051726 | regulation of cell cycle | 17 | 0.006 | 1 | 0.016 |
| GO:0071363 | cellular response to growth factor stimulus | 19 | 0.031 | 1 | 0.037 |
| GO:0071637 | regulation of monocyte chemotactic protein-1 production | 2 | 0.032 | 1 | 0.037 |
| GO:0071702 | organic substance transport | 83 | 0.000 | 0.007 | 0.000 |
| GO:0071840 | cellular component organization or biogenesis | 140 | 0.012 | 1 | 0.024 |
| GO:0090304 | nucleic acid metabolic process | 131 | 0.011 | 1 | 0.023 |
| GO:1901576 | organic substance biosynthetic process | 153 | 0.005 | 1 | 0.014 |
| GO:0009057 | macromolecule catabolic process | 48 | 0.000 | 0.036 | 0.000 |
| GO:0009607 | response to biotic stimulus | 25 | 0.000 | 0.406 | 0.004 |
| GO:0009611 | response to wounding | 38 | 0.034 | 1 | 0.039 |
| GO:0009891 | positive regulation of biosynthetic process | 46 | 0.021 | 1 | 0.033 |
| GO:0009896 | positive regulation of catabolic process | 8 | 0.028 | 1 | 0.036 |
| GO:0009914 | hormone transport | 12 | 0.032 | 1 | 0.037 |
| GO:0010604 | positive regulation of macromolecule metabolic process | 69 | 0.003 | 1 | 0.011 |
